# Supplementary material for: An RNA-Scaffold Protein Subunit Vaccine for Nasal Immunization
Source: Vaccines (Basel). 2023 Sep 29;11(10):1550. doi: 10.3390/vaccines11101550 (PMC10610892; doi:10.3390/vaccines11101550)
Supplement: Supplementary file 1 [file vaccines-11-01550-s001.zip › vaccines-2626938-supplementary.pdf]

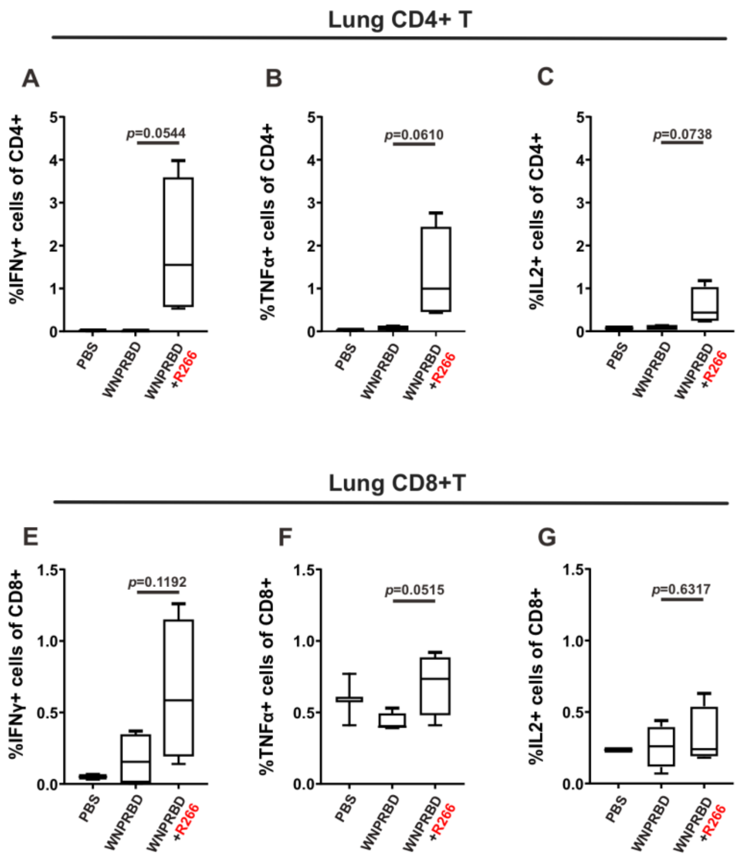

Supplementary figure 1. WNPRBD-R266 activates T cell responses in mice lung. Lung of the same mice described in T cell assays in Figure 4 were also harvested and analyzed for lung T cell activation. (A-C) CD4+ and (D-F) CD8+ antigen-specific T cells were analyzed.

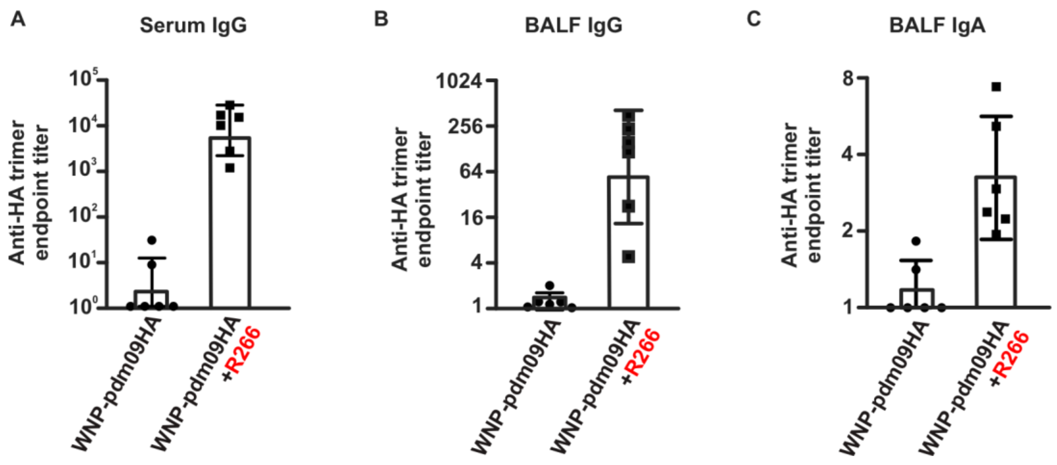

Supplementary figure 2. Ribonucleoprotein influenza A-H1N1 version elicits mucosal and neutralizing antibodies in mice. (A-C) BALB/c mice were intranasally immunized with 2 doses of WNP-pdm09HA-R266, with 14 days in between. Sera and BALF were harvested at 14 days post 2nd dose. HA-trimer specific antibody levels were determined by ELISA.
